# Low utilization of glucose in the liver causes diet-induced hypercholesterolemia in exogenously hypercholesterolemic rats

**DOI:** 10.1371/journal.pone.0229669

**Published:** 2020-03-12

**Authors:** Yasutake Tanaka, Masahiro Ono, Motonori Miyago, Takahisa Suzuki, Yurika Miyazaki, Michio Kawano, Makoto Asahina, Bungo Shirouchi, Katsumi Imaizumi, Masao Sato

**Affiliations:** Department of Bioscience and Biotechnology, Laboratory of Nutrition Chemistry, Faculty of Agriculture, Graduate School, Kyushu University, Fukuoka, Japan; University of Illinois, UNITED STATES

## Abstract

Exogenously hypercholesterolemic (ExHC) rats develop diet-induced hypercholesterolemia (DIHC) when fed with dietary cholesterol. Previously, we reported that, under the high-sucrose-diet-feeding condition, a loss-of-function mutation in *Smek2* results in low activity of fatty acid synthase (FAS) followed by the shortage of hepatic triacylglycerol content in ExHC rats and the onset of DIHC. However, the relationship between the *Smek2* mutation and FAS dysfunction is still unclear. Here, we focused on carbohydrate metabolism, which provides substrates for FAS, and analyzed carbohydrate and lipid metabolisms in ExHC rats to clarify how the deficit of *Smek2* causes DIHC. Male ExHC and SD rats were fed high-sucrose or high-starch diets containing 1% cholesterol for 2 weeks. Serum cholesterol levels of the ExHC rats were higher, regardless of the dietary carbohydrate. Hepatic triacylglycerol levels were higher in only the SD rats fed the high-sucrose diet. Moreover, the ExHC rats exhibited a diabetes-like status and accumulation of hepatic glycogen and low hepatic mRNA levels of liver-type phosphofructokinase (*Pfkl*), which encodes a rate-limiting enzyme for glycolysis. These results suggest that the glucose utilization, particularly glycolysis, is impaired in the liver of ExHC rats. To evaluate how the diet with extremely low glucose affect to DIHC, ExHC.BN-*Dihc2*^*BN*^, a congenic strain that does not develop DIHC, and ExHC rats were fed a high-fructose diet containing 1% cholesterol for 2 weeks. The serum cholesterol and hepatic triacylglycerol levels were similar in the strains. Results of water-soluble metabolite analysis with primary hepatocytes, an increase in fructose-6-phosphate and decreases in succinate, malate and aspartate in ExHC rats, support impaired glycolysis in the ExHC rats. Thus, the *Smek2* mutation causes abnormal hepatic glucose utilization via downregulation of *Pfkl* expression. This abnormal glucose metabolism disrupts hepatic fatty acid synthesis and causes DIHC in the ExHC rats.

## 1. Introduction

For decades, hypercholesterolemia has been reported as a risk factor for arterial sclerosis and cardiac infarction. Serum cholesterol levels are determined by the balance of cholesterol uptake from the blood and cholesterol synthesis in the whole body, which is regulated by gene expressions and dietary cholesterol intake. Many studies have reported lifestyle (dietary and daily habits) and genetic mutations (SNPs, loss-of-function, or gain-of-function mutations) as risk factors for hypercholesterolemia [1–6]. For instance, a mutation in PCSK9 causes degradation of the low-density lipoprotein receptor to increase blood cholesterol levels [6]. On the other hand, the contribution of dietary cholesterol intake in the determination of serum cholesterol levels has been re-considered. The Ministry of Health, Labor and Welfare in Japan discontinued the upper limit of dietary cholesterol intake [7] after a similar announcement by the United States Department of Agriculture and United States Department of Health and Human Services [8]. However, this does not mean that the dietary cholesterol intake does not affect serum cholesterol levels. Katan et al. reported hyper- and hypo-responders to dietary cholesterol [9]. The serum cholesterol levels of hyper-responders after a high-cholesterol diet period were significantly higher than those of hypo-responders [9]. In addition, several studies have implied that cholesterol metabolism is affected by the metabolism of other nutrients [10,11]. Hypercholesterolemia is frequently present in metabolic syndrome and type 2 diabetes [11,12]. Hypercholesterolemia should be considered as a complex disorder of cholesterol metabolism as well as the metabolism of multiple nutrients.

Exogenously hypercholesterolemic (ExHC) rats, originally derived from Sprague-Dawley (SD) rats, develop diet-induced hypercholesterolemia (DIHC) only when fed with dietary cholesterol [13]. In our previous studies, we performed linkage analysis of ExHC rats and detected two genetic loci, Dihc1 on chromosome 5 and Dihc2 on chromosome 14 [14]. Furthermore, loss-of-function mutation (10-bp-deletion) on *Smek2* in the Dihc2 region was found to be attributable to the DIHC [15]. To reveal the mechanism underlying the development of DIHC, we performed comparative studies with ExHC, SD, and ExHC.BN-*Dhic2*^*BN*^ congenic rats (described as Congenic rats in the following text), which have the Dich2 locus derived from Brown-Norway (BN) rats, used as a genetic standard strain, and genetic background of ExHC rats [15]. Under the sucrose-diet-fed condition activating hepatic *de novo* fatty acid synthesis, the ExHC rats showed low hepatic fatty acid synthase (FAS) activity and low hepatic triacylglycerol (TAG) levels because of *Smek2* dysfunction [16]. The shortage of hepatic TAG resulted in the retention of very low-density lipoprotein (VLDL) via β-migration (increase in cholesterol-ester population in secreted VLDL) [16]. However, the mechanism underlying the depression of hepatic FAS activity caused by *Smek2* dysfunction is still unclear. In this study, we focused on and evaluated carbohydrate metabolism in ExHC rats. Carbohydrate metabolism supplies substrates for hepatic lipid synthesis. In addition, Yoon et al. reported that SMEK acts as a subunit of protein phosphatase 4 (PP4) [17] and upregulates gluconeogenesis in human hepatocytes [17]. PP4 has also been reported to regulate insulin resistance induced by tumor necrosis factor-α [18] and promotes lipid synthesis by dephosphorylation and activation of acetyl CoA carboxylase 1 [19].

In this study, we conducted two experiments with three types of diet, different ratios of carbohydrates (glucose, sucrose, and fructose). As we reported, ExHC rats show low FAS activities even though being fed the high-sucrose diet [16]. Therefore, we conducted the experiment 1 with a hypothesis that ExHC rats had an impaired metabolism of fructose, which can skip the rate-limiting reaction of glycolysis and be more rapidly metabolized than glucose. Additionally, we also performed the experiment 2 for the comparison of ExHC and congenic rats under the high-fructose-diet feeding condition. In experiment 2, congenic rats were used to clearly observe the effect of *Smek2*. On the basis of the results, we evaluated the relationship between glucose and lipid metabolism in ExHC rats and revealed the pathological mechanism underlying DIHC in ExHC rats.

## 2. Materials and methods

### 2.1 Animals and diet

Colonies of male ExHC and ExHC.BN-*Dihc2*^*BN*^ rats are being maintained at our institute (Kyusyu University, Faculty of agriculture, Laboratory of nutrition chemistry) through brother-sister inbreeding. Male 25-day-old SD/Kud rats were purchased from Kyudo Co., Ltd. (Saga, Japan). These animals had free access to a commercial non-purified diet (NMF; Oriental Yeast Co., Ltd., Tokyo, Japan) and deionized water, and they were maintained in a temperature-controlled room at 22–25°C with a 12-h light cycle (08:00–20:00) for 3 days. In experiment 1, ExHC and SD rats were fed a high-sucrose or high-starch diet and divided into 4 groups each (n = 5/group). In Experiment 2, ExHC and ExHC.BN-*Dhic2*^*BN*^ rats were fed a high-fructose diet. ExHC and ExHC.BN-*Dhic2*^*BN*^ rats for primary hepatocytes were fed the NMF diet until fasting before the operation. The experimental diets were formulated according to the AIN76™ formula (High-sucrose diet; 44% sucrose, 15% corn starch, High-sucrose diet; 9% sucrose, 50% corn starch, High fructose diet; 9% sucrose, 50% fructose, the details of dietary ingredients are represented as S1 Table). On day 8, the oral glucose tolerance test (OGTT) was performed. After consuming the experimental diets for 2 weeks (6 weeks of age), the rats were killed by decapitation after 9 h of fasting under pentobarbital anesthesia. Blood samples were collected and maintained at room temperature for 30 min and then centrifuged at 1,750 × *g* and 4°C for 15 min to collect the serum. The livers were immediately excised, weighed, and frozen in liquid nitrogen and maintained at −30°C until analysis. To minimize the animal pain, the handling and killing of all the animals were performed in accordance with nationally prescribed guidelines, and ethical approval for the experiments was granted by the Animal Care and Use Committee, Kyushu University (Scientific Research Promotion Division; authorization number: A26-098-3).

### 2.2 OGTT

OGTT was performed on day 8 of experiment 1. The rats were subjected to 16 h of fasting and then orally administrated a glucose bolus (3 g/kg body weight). Blood samples were obtained from the tail vein at 0, 15, 30, 45, 60, 75, 90, 120, 180, and 240 min after the glucose load. Blood glucose concentrations were measured using an Accu-Chek^®^ Aviva Nano blood glucose meter (Roche Diagnostics, Tokyo, Japan). Blood samples at each time point were collected in heparinized microhematocrit capillaries (HIRSCHMANN^®^; Hirschmann Laborgeräte GMbH & Co., Eberstadt, Germany) and centrifuged at 1,750 × *g* and room temperature for 15 min to obtain the plasma. Plasma insulin levels were measured with the Rat Insulin ELISA kit (Shibayagi, Gunma, Japan). Area under the curve (AUC) and incremental area under the curve (iAUC) were calculated from the glucose and insulin levels at each point.

### 2.3 Analysis of serum and liver parameters

Serum levels of cholesterol, TAG, non-esterified fatty acid (NEFA, free fatty acid), glucose, phospholipid, and free glycerol were measured with enzyme assay kits (Cholesterol E-Test, Triglyceride E-Test, NEFA C-Test, Phospholipid C-test, Glucose CII-Test: Wako Pure Chemical Industries, Osaka, Japan; Glycerol Assay Kit: Cayman Chemical Company, Ann Arbor, USA). Homeostasis model assessment as an index of insulin resistance (HOMA-IR) was calculated using the following formula [20].

$${\text{HOMA-IR}=\text{G}}_{\text{fast}}{\times \text{I}}_{\text{fast}}/405$$

$$\left({\text{G}}_{\text{fast}}:\text{Fasting Glucose levels}\;\left[\text{mg/dL}\right]{,\;\text{I}}_{\text{fast}}:\;\text{Fasting insulin level}\;\left[\mu \text{U/mL}\right]\right)$$

### 2.4 Determination of hepatic FAS

Two grams of liver was homogenized in six volumes of an ice-cold homogenize buffer containing 0.25 M sucrose, 1 mM EDTA, and 10 mM Tris–HCl (pH 7.4). After the nucleus fraction was precipitated, the supernatant was centrifuged at 10,000 × *g* and 4°C for 10 min to separate the mitochondrial fraction. The resulting supernatant was re-centrifuged at 125,000 × *g* and 4°C for 60 min to precipitate the microsomes, and the remaining supernatant was used as the cytosol fraction. The protein levels were determined according to the method by Lowry et al. [21], and BSA was used as the standard. The enzyme activities of FAS in the cytosol fraction were determined using Buang’s method [22].

### 2.5 Determination of hepatic mRNA levels

Total cellular RNA was isolated from the liver tissue by using the phenol/chloroform method [23]. Complementary DNA (cDNA) was synthesized from 1.0 μg of the total RNA by using a Transcriptor First Strand cDNA Synthesis Kit (Roche, Berlin, Germany). The expression level for phosphofructokinase (*Pfkl*) was analyzed using quantitative real-time reverse transcription polymerase chain reaction (RT-PCR) with a SYBR Premix EX Taq II kit and Thermal Cycler Dice Real Time System TP800 (TaKaRa, Shiga, Japan). The mRNA levels were normalized using the β-actin gene (*Actb*) as an internal standard. The primer sequences used for the analysis were as follows: *Pfkl* (F): 5′-CCTTTGTGTTGGAGGTGATG-3′, *Pfkl* (R): 5′-GATGATGTTCAGTCGAGACC-3′, *Actb* (F): 5′-TCAGGTCATCATCACTATCGGCA-3′, *Actb* (R): 5′-TCATGGATGCCACAGGATTC-3′.

### 2.6 Sampling of rat primary hepatocyte

The livers of the ExHC and Congenic rats (n = 4/strain) fasted for 24 h were perfused from the portal vein to the right atrium with ethylene glycol tetraacetic acid (EGTA) solution (S2 Table) and collagenase solution (S3 Table) under anesthesia. The livers were collected in collagenase solution and incubated at 37°C for 10 min. After filtration with gauze, the collected hepatocytes were washed 3 times by centrifugation (50 × *g* for 2 min) and re-suspension in Hanks’ solution (S4 Table). The hepatocytes were resuspended in Williams medium E (WE medium) containing 10% FBS at 5 × 10^5^ cells/ml and seeded into a collagen-coated dish. After 3 h of incubation at 37°C, the medium was exchanged to remove dead hepatocytes. After 24 h of incubation, the primary hepatocytes were used for further experiments.

### 2.7 Analysis of water-soluble metabolites in the rat primary hepatocyte (non-target metabolome analysis)

Water-soluble metabolites in the rat primary hepatocyte lysate were analyzed according to a previously described protocol [24]. Same-cell concentrations of primary hepatocytes of ExHC and Congenic rats were collected in PBS and then sonicated to obtain the cell lysate. This lysate (50 μl) was used for the metabolome analysis performed as described previously [24]. Metabolites extracted from cell lysate were derivatized with Methoxyamine hydrochloride and N-Methyl-N-trimethylsilyl-trifluoroacetamide and detected with gas chromatography mass spectroscopy analysis (GCMS-QP2020, SHIMAZU). The digitalization of each peak and identification of metabolites data were operated with free analytical softwares (MetAlign, MetaboAnalyst). The values of each metabolite were adjusted as relative to the internal standard. From all identified metabolites, data of metabolites were extracted under the following conditions.

1. Average intensity of QC (measured by mixing all samples) is 1000 or more
2. Coefficient of Variation (CV) value of QC is 100% or less
3. Blank (solvent only) intensity is 20% of QC average intensity or less
4. CV value within the group is 150% or less

### 2.8 Statistical analysis

All values are expressed as means and standard errors of means (SEM). In OGTT, blood glucose and plasma insulin levels were analyzed using Student’s *t*-test between strains in the same diet groups. The other data were analyzed using two-way analysis of variance (ANOVA), and the differences were considered statistically significant when *p* was <0.05. When interactions were detected using two-way ANOVA, the data were reanalyzed with the Tukey-Kramer multiple comparison post-hoc test, and the differences were considered statistically significant at *p* <0.05. The statistical analyses were performed using Excel 2011 (Microsoft, USA) with the add-in software Statcel 3.

## 3. Results

### Experiment 1

#### 3.1 Growth parameters and organ weights

Table 1 shows the growth and serum biochemical parameters in Experiment 1. The weights of the brain, kidney, and total white adipose tissue (WAT) of the ExHC rats were significantly heavier. The weights of the femoral muscle of the ExHC rats were significantly lighter than those of the SD rats. However, consumption of the high-starch diet resulted in a significant increase in the brain weight. The pancreas and kidney weights were significantly decreased by the high-starch diet. No significant differences were found in the other growth parameters.

**Table 1:** Growth parameters, organ weights, and biochemical parameters in Experiment 1.

	High-sucrose diet				High-starch diet				Strain	Carbo-hydrate	S×C
	SD		ExHC		SD	ExHC					
Growth parameters											
Initial body weight(g)	89.7 ± 3.8		85.3 ± 8.5		76.2 ± 0.8		76.1 ± 1.4		N.S.	*P<0*.*05*	N.S.
Body weight gain(g)	78.2 ± 3.8		83 ± 2.5		77.1 ± 4.1		73 ± 3.2		N.S.	N.S.	N.S.
Food intake(g)	203 ± 10		204 ± 10		188 ± 1		187 ± 2		N.S.	*P<0*.*05*	N.S.
Food efficiency (g body weight gain/ g food intake)	0.386 ± 0.015		0.409 ± 0.012		0.411 ± 0.02		0.391 ± 0.02		N.S.	N.S.	N.S.
Organ weights	*g organ weight/100g body weight*										
Liver	5.16 ± 0.30		4.85± 0.16		4.88 ± 0.07		5.04 ± 0.07		N.S.	N.S.	N.S.
Brain	0.72 ± 0.02		0.77 ± 0.04		0.82 ± 0.02		0.89 ± 0.02		*P<0*.*05*	*P<0*.*01*	N.S.
Pancreas	0.27 ± 0.02		0.32 ± 0.03		0.25 ± 0.01		0.22 ± 0.02		N.S.	*P<0*.*05*	N.S.
Kidney	0.95 ± 0.01		1.01 ± 0.01		0.88 ± 0.02		0.93 ± 0.02		*P<0*.*01*	*P<0*.*01*	N.S.
Femoral muscle	1.22 ± 0.03		1.08 ± 0.01		1.21 ± 0.02		1.03 ± 0.03		*P<0*.*01*	N.S.	N.S.
Total White adipose tissue	3.95 ± 0.26		4.89 ± 0.24		4.39 ± 0.16		4.94 ± 0.11		*P<0*.*01*	N.S.	N.S.
Brown adipose tissue	0.13 ± 0.01		0.14 ± 0.01		0.16 ± 0.01		0.15 ± 0.01		N.S.	0.075	N.S.
Biochemical parameters											
Serum											
Free cholesterol (mg/dL)	22.1 ± 1.8		116 ± 5		23.6 ± 4.5		108 ± 13		*P<0*.*01*	N.S.	N.S.
Cholesterol ester (mg/dL)	88.7 ± 12.6		381 ± 12		165 ± 24		462 ± 42		*P<0*.*01*	*P<0*.*01*	N.S.
Free Fatty Acid (mEq./L)	0.90 ± 0.03	b	1.65 ± 0.05	c	0.35 ± 0.02	a	0.49 ± 0.06	a	*P<0*.*01*	*P<0*.*01*	*P<0*.*01*
Phospholipids (mg/dL)	130 ± 7		226 ± 7		146 ± 5		256 ± 22		*P<0*.*01*	0.078	N.S.
Free glycerol (mg/dL)	1.94 ± 0.17		2.61 ± 0.1		2.26 ± 0.24		3.39 ± 0.29		*P<0*.*01*	*P<0*.*05*	N.S.
HOMA-IR	1.05 ± 0.41	a	3.85 ± 0.7	b	1.21 ± 0.15	a	1.93 ± 0.39	ab	*P<0*.*01*	0.069	*P<0*.*05*

Values are means ± standard errors of the means (SEMs). n = 5/group. The data were analyzed with two-way ANOVA, followed by the Tukey-Kramer test as a post-hoc test. a,b: Different superscripts show significant differences at *P* < 0.05. *N*.*S*.: not significant.

#### 3.2 Serum parameters

**Fig 1:**
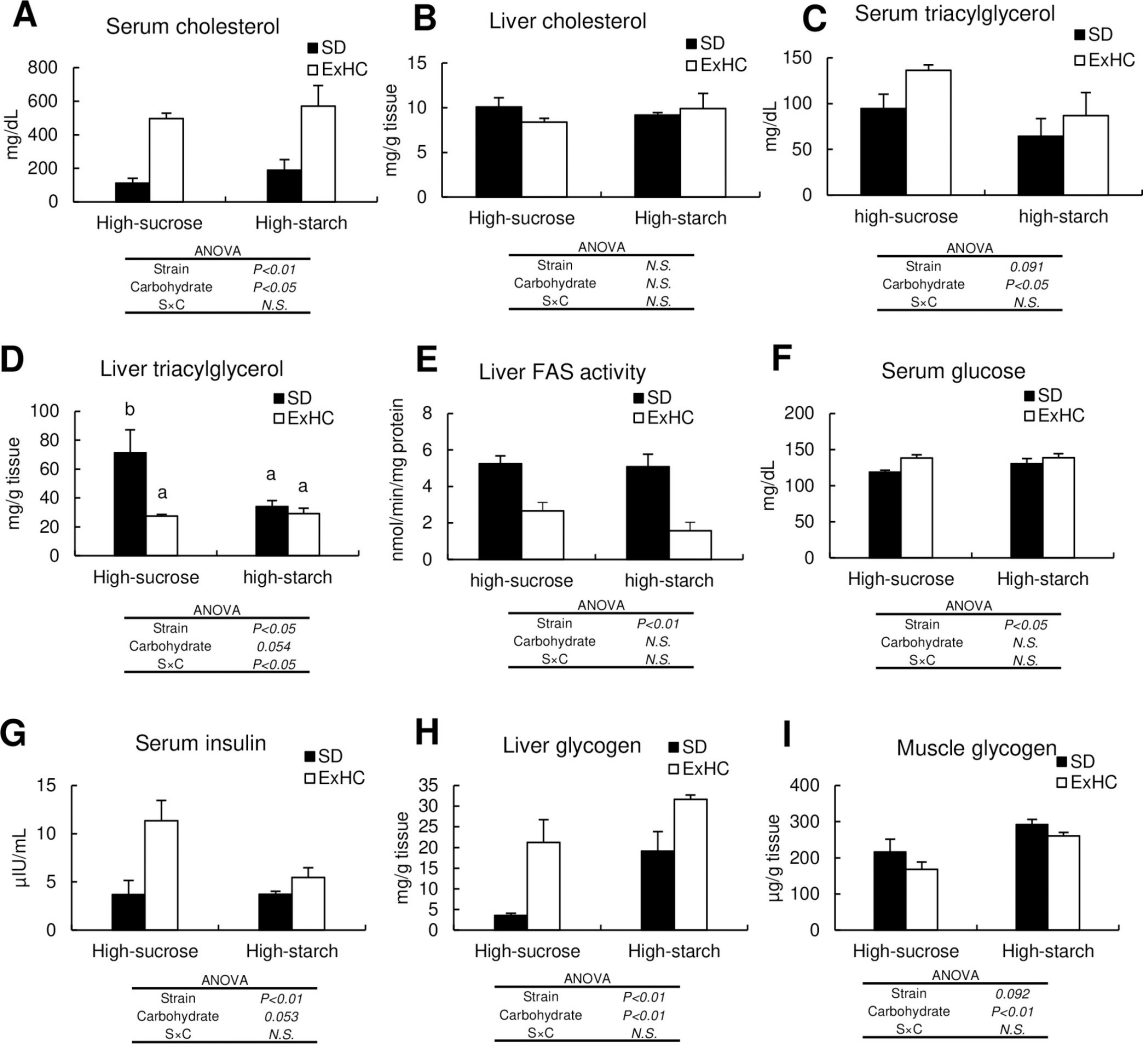
Biochemical parameters in Experiment 1. (A) serum cholesterol, (B) liver cholesterol, (C) serum triacylglycerol, (D) liver triacylglycerol, (E) liver fatty acid synthase activity, (F) serum glucose, (G) serum insulin, (H) liver glycogen, (I) muscle glycogen. Values are means ± standard errors of the means (SEMs). Solid bar and open bar represent the data of SD, ExHC, respectively. n = 5/group. The data were analyzed with two-way ANOVA, followed by the Tukey-Kramer test as a post-hoc test. a,b: Different superscripts show significant differences at *P* < 0.05. *N*.*S*.: not significant.

The serum lipid parameters of the ExHC rats measured in Experiment 1 were significantly higher (Table 1). When compared with the high-sucrose diet, the high-starch diet significantly increased the serum levels of total cholesterol (Fig 1A), cholesterol ester, and free glycerol and significantly decreased the serum TAG levels. In the serum NEFA levels, an interaction between strain and diet was observed. The high-sucrose-ExHC group showed the highest levels of serum NEFA. Among the rest, the high-sucrose-SD group showed higher serum NEFA levels than the high-starch-SD and high-starch-ExHC groups.

The serum fasting glucose and insulin levels were significantly higher in the ExHC rats, regardless of the diet (Fig 1F and 1G). The values of HOMA-IR in the high-sucrose-ExHC group were the highest in Experiment 1 (Table 1).

#### 3.3 Organ biochemical parameters

The ExHC rats showed significantly higher hepatic glycogen levels and tended to have lower muscular glycogen levels (Fig 1H and 1I). These glycogen levels were significantly increased by consumption of the high-starch diet. With respect to the hepatic TAG levels, an interaction between strain and diet was observed (Fig 1D). The hepatic TAG levels of the high-sucrose-SD group were the highest. The hepatic FAS activities in the ExHC rats were significantly lower than those in the SD rats in both diet groups (Fig 1E).

#### 3.4 OGTT

**Fig 2:**
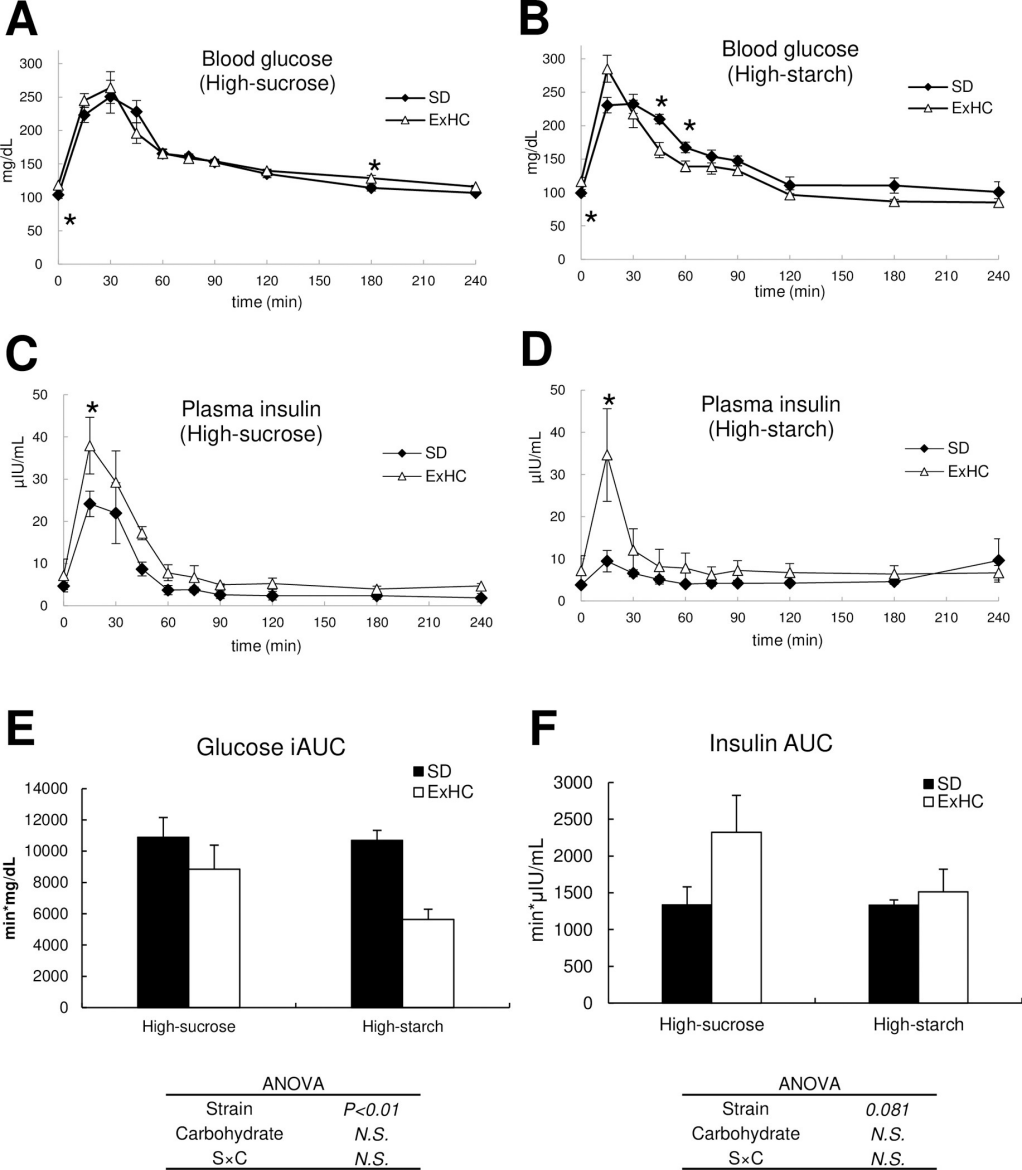
Results of the oral glucose tolerance test. (A–B) Blood glucose levels and (C–D) plasma insulin levels after glucose bolus (SD: open square, ExHC: solid triangle). (E) Incremental areas under the time-concentration curve (AUC) for blood glucose. (F) AUCs for serum insulin. Values are means ± standard errors of the means (SEMs). n = 5/group. The data between strains at the same time after glucose bolus were analyzed using Student’s *t*-test (A–D) or two-way ANOVA followed by the Tukey-Kramer test (E–F). *: asterisks show significant differences at *P* < 0.05. a,b: Different superscripts show significant differences at *P* < 0.05. *N*.*S*.: not significant.

The initial serum glucose levels of the ExHC rats were significantly higher than those of the SD rats in spite of the diets (Fig 2A and 2B). When fed the high-sucrose diet, the ExHC rats showed significantly higher blood glucose levels at 180 min after glucose loading (Fig 2A). However, the high-starch-ExHC group showed significantly lower blood glucose levels at 45 min and 60 min (Fig 2B). Glucose iAUCs in the ExHC rats were significantly lower than those of the SD rats in both diet groups (Fig 2E). At 15 min after glucose administration, plasma insulin levels of the ExHC rats were significantly higher than those of the SD rats (Fig 2C and 2D). The ExHC rats showed a decreasing tendency with respect to insulin AUCs (Fig 2F).

#### 3.5 Hepatic glycolysis gene expressions

**Fig 3:**
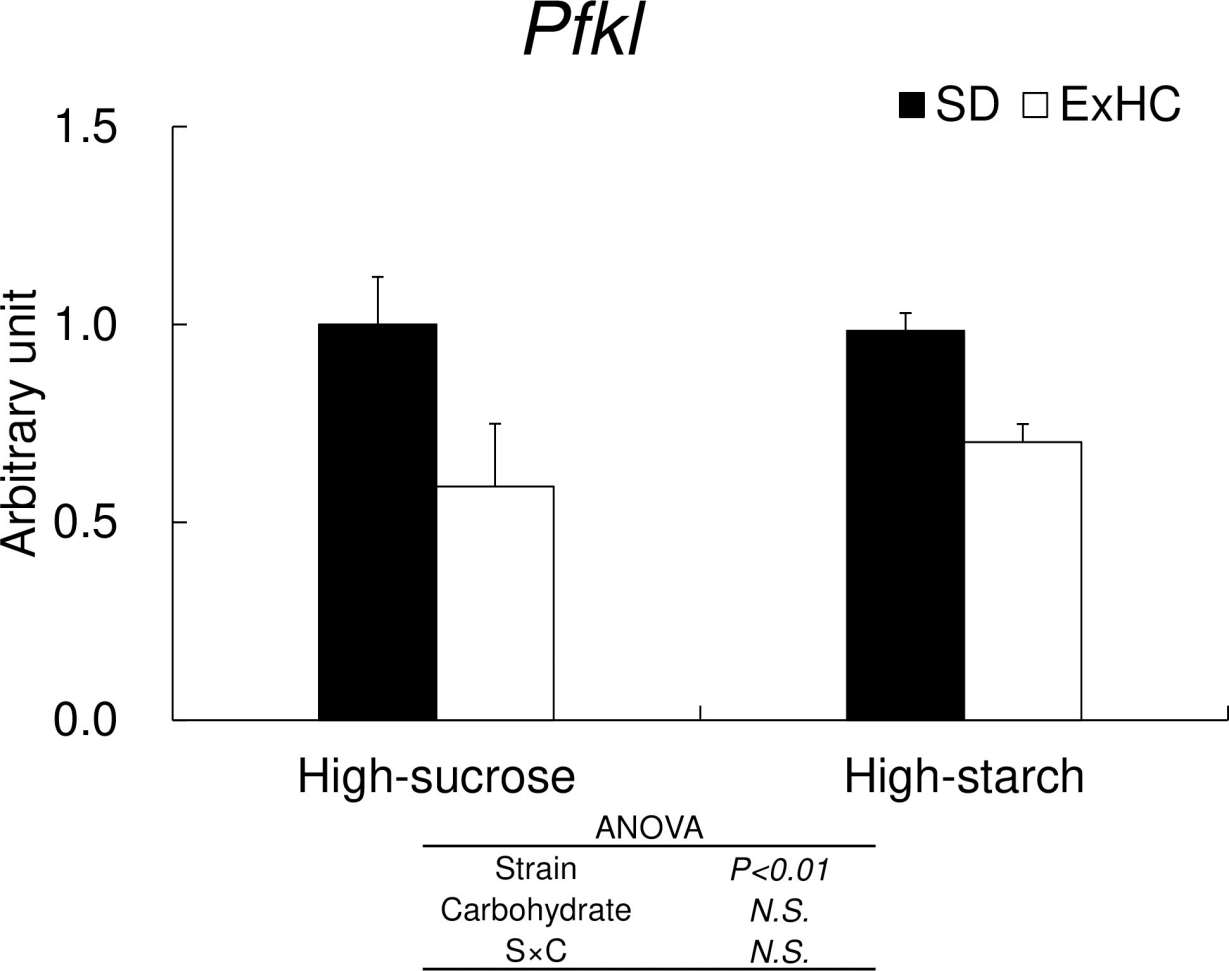
*Pfkl* hepatic mRNA expressions in Experiment 1. Hepatic *Pfkl* mRNA expressions were measured using real-time RT-PCR (SD: solid bar, ExHC: open bar). Values are means ± standard errors of the means (SEMs). n = 5/group. The data for were analyzed with two-way ANOVA, followed by the Tukey-Kramer test as a post-hoc test. *N*.*S*.: not significant.

The mRNA levels of *Pfkl* in the liver were significantly lower in the ExHC rats than in the SD rats (Fig 3). No effects of the diets on hepatic *Pfkl* mRNA levels were observed.

### Experiment 2

#### 3.6 Growth parameters and biochemical parameters

**Fig 4:**
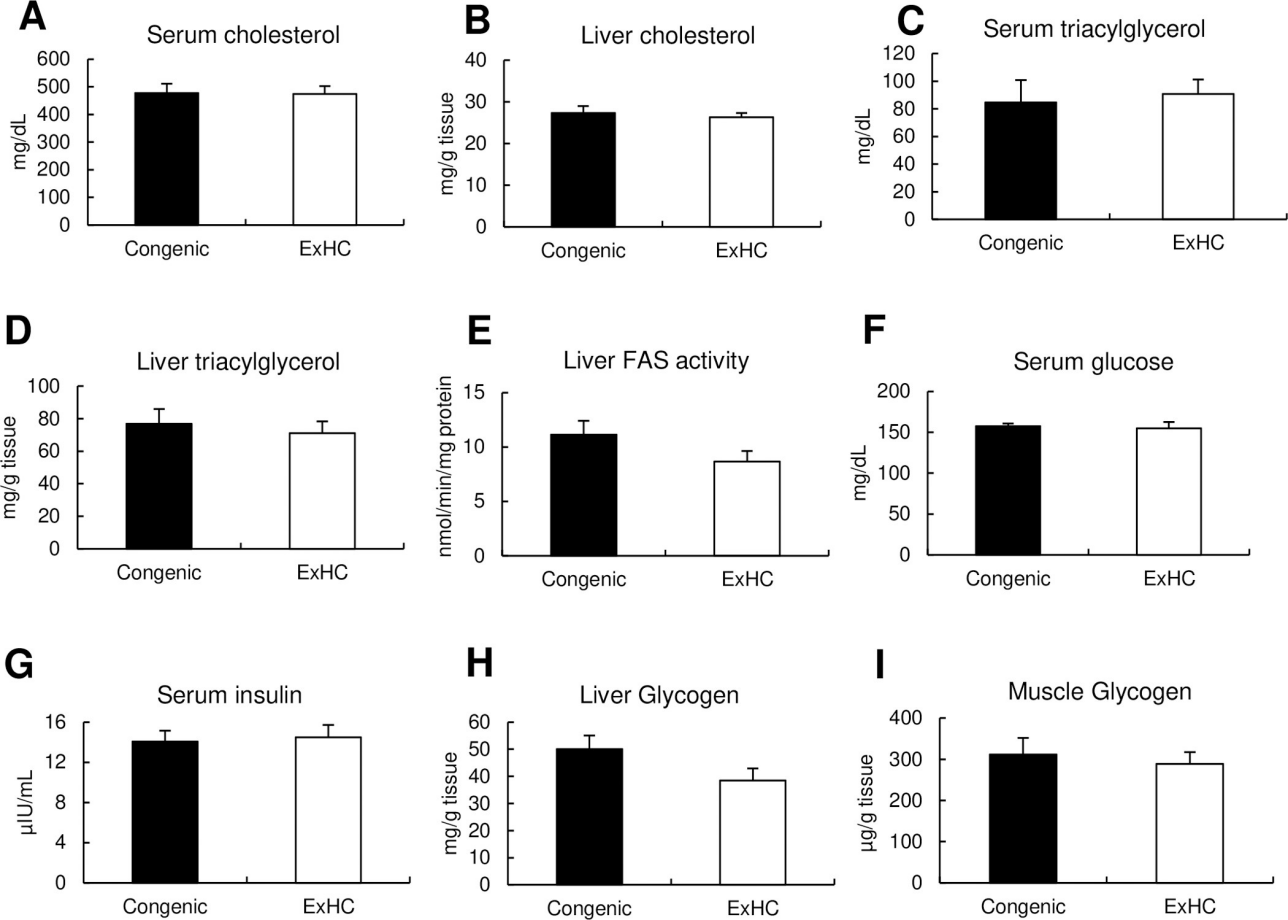
Biochemical parameters directly related to DIHC in Experiment 2. (A) serum cholesterol, (B) liver cholesterol, (C) serum triacylglycerol, (D) liver triacylglycerol, (E) liver fatty acid synthase activity, (F) serum glucose, (G) serum insulin, (H) liver glycogen, (I) muscle glycogen. Values are means ± standard errors of the means (SEMs). Solid bar and open bar represent the data of SD, ExHC, respectively. n = 5/group. The data were analyzed with Student’s *t*-test.

After the consumption of the high-fructose diet, no significant differences in parameters related to the development of DIHC were observed (Fig 4). The brain weights, total WAT, and BAT of the Congenic rats were significantly higher than those of the ExHC rats (Table 2). The femoral muscle weights of the Congenic rats were significantly lower than those of the ExHC rats (Table 2). The biochemical parameters of the serum and organs were similar between the strains (Table 2).

**Table 2:** Growth parameters, organ weights, and biochemical parameters after the high-fructose diet.

	ExHC (Smek2-)	Congenic (Smek2+)	Statistics
Growth parameters			
Initial body weight (g)	77.1 ± 1.1	78.5 ± 1.9	
Final body weight (g)	138.6 ± 3.1	135.6 ± 3.2	
Body weight gain (g)	56.8 ± 2.2	58.5 ± 3.4	
Food intake (g)	163 ± 3	164 ± 2	
Food efficiency (g body weight gain/g food intake)	0.350 ± 0.015	0.357 ± 0.016	
Organ weights	*g organ weight/100g body weight*		
Liver	5.39 ± 0.08	5.65 ± 0.22	
Brain	0.900 ± 0.020	0.970 ± 0.020	*
Pancreas	0.480 ± 0.010	0.480 ± 0.010	
Kidney	1.14 ± 0.01	1.15 ± 0.02	
Femoral muscle	1.13 ± 0.09	0.87 ± 0.07	*
Total white adipose tissue	4.93 ± 0.28	6.56 ± 0.32	*
Brown adipose tissue	0.130 ± 0.010	0.180 ± 0.010	*
Biochemical parameters			
Serum			
Free cholesterol (mg/dL)	85.5 ± 10.5	83.2 ± 7.7	
Cholesterol ester (mg/dL)	389 ± 29	394 ± 40	
Free Fatty Acid (mEq./L)	0.904 ± 0.075	0.864 ± 0.132	
HOMA-IR	5.53 ± 0.53	5.45 ± 0.39	

Values are means ± standard errors of the means (SEMs). n = 5/group. The data were analyzed using Student’s *t*-test.

*: asterisks show significant differences at *P* < 0.05.

#### 3.7 Hepatic glycolysis gene expressions

**Fig 5:**
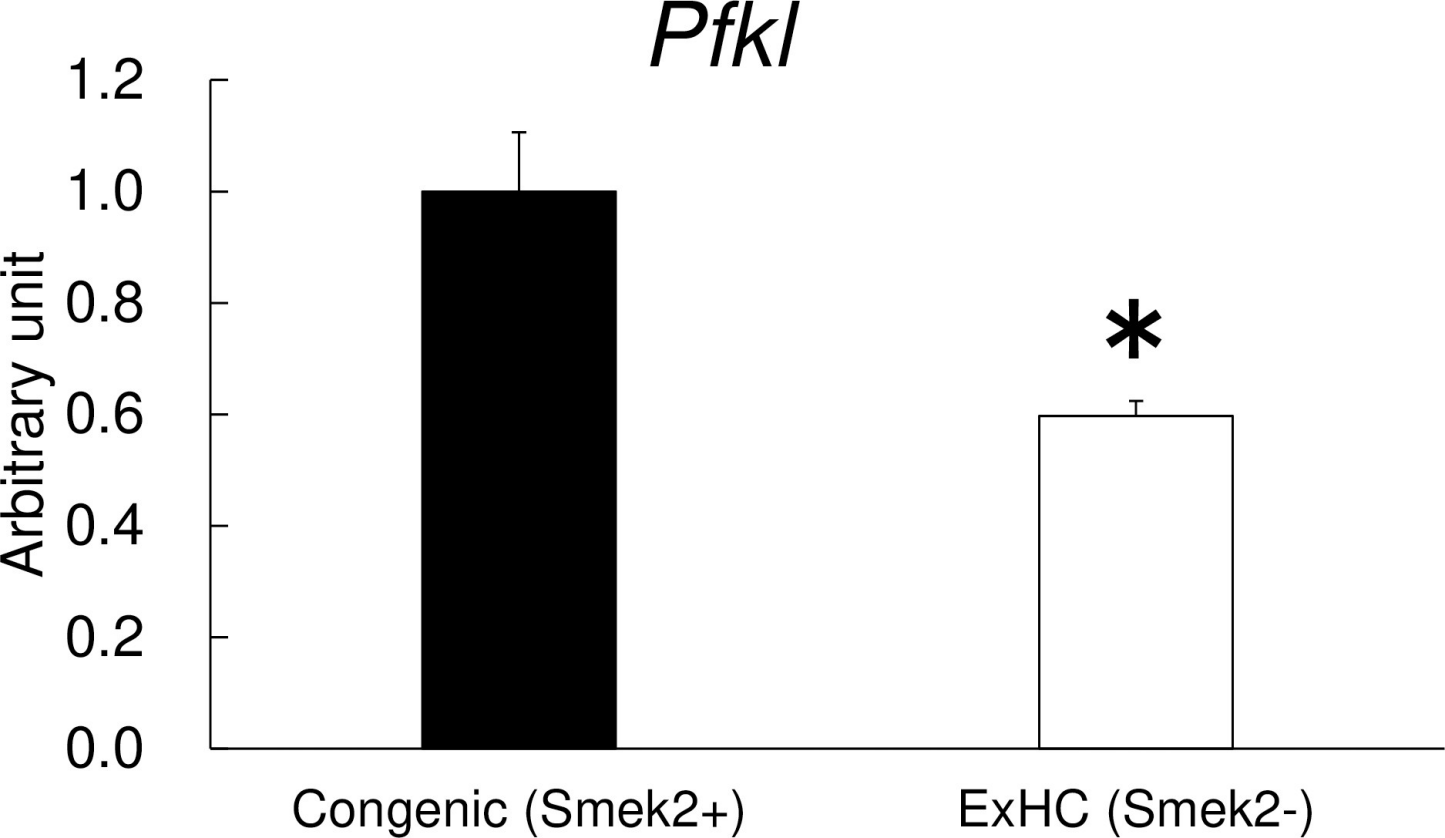
*Pfkl* hepatic mRNA expressions after the high-fructose diet. Hepatic *Pfkl* mRNA expressions were measured using real-time RT-PCR (Congenic: solid bar, ExHC: open bar). Values are means ± standard errors of the means (SEMs). n = 5/group. The data were analyzed using Student’s *t*-test. *: asterisks show significant differences at *P* < 0.05.

Hepatic *Pfkl* mRNA levels in the Congenic rats were significantly higher than those in the ExHC rats (Fig 5).

#### 3.8 Analysis of water-soluble metabolites in the rat primary hepatocytes

**Fig 6:**
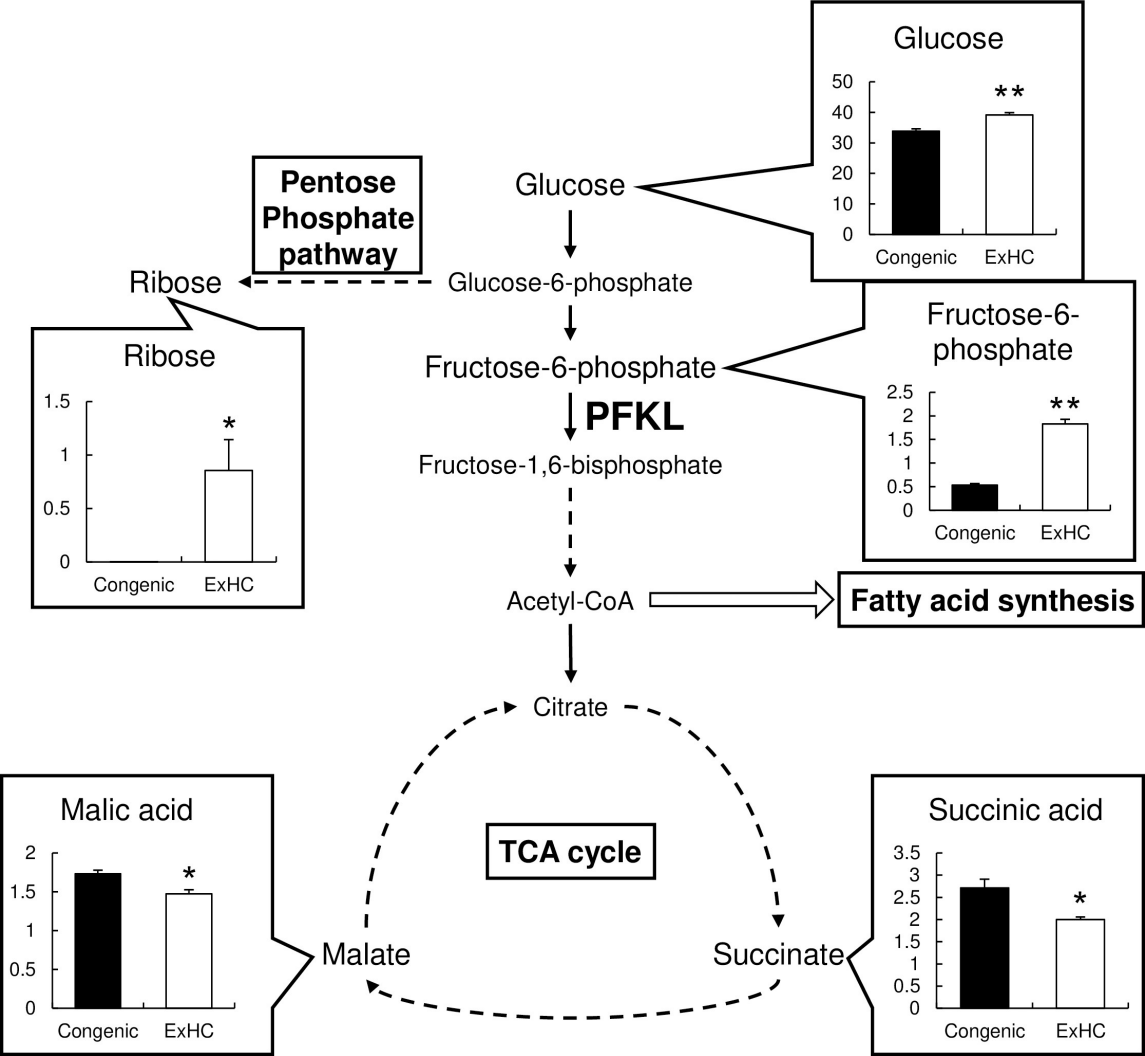
Water-soluble metabolites related to cellular glucose metabolism in rat primary hepatocytes. The water-soluble metabolites were analyzed with primary hepatocytes of ExHC and ExHC.BN-*Dihc2*^*BN*^ rats. The solid and dashed arrows represent direct and multistep reactions, respectively. Values are means ± standard errors of the means (SEMs). n = 4/group. The data were analyzed using Student’s *t*-test. *, **: asterisks show significant differences at *P* < 0.05 and 0.01, respectively.

As a result of GC-MS analysis, 153 compounds including duplicates and unknowns were detected. Of these, 25 compounds were extracted by selection based on detection intensity and dispersion (Table 3). When compared with the primary hepatocytes of the Congenic rats, 6 metabolites (fructose 6-phosphate, glucose, ribose, trehalose, leucine, and tyramine) were increased and another 6 metabolites (aspartic acid, glycine, 4-aminobutylic acid, malic acid, succinic acid, and urea) were significantly decreased in the hepatocytes of the ExHC rats (Fig 6 and Table 3).

**Table 3:** Water-soluble metabolite analysis with primary hepatocytes of ExHC and congenic rats.

Metabolite	Congenic (*Smek2+*)	ExHC (*Smek2-*)	*Statistics*
Sugar	*Relative Intensity (internal std*. *= 100)*		
Fructose 6-phosphate	0.532 ± 0.034	1.83 ± 0.10	**
Glucose	33.9 ± 0.7	39.2 ± 0.7	**
Mannose	0.453 ± 0.039	0.495 ± 0.035	
Ribose	0.002 ± 0.000	0.856 ± 0.288	*
Trehalose	2.20 ± 0.05	2.67 ± 0.18	*
Amino acid			
Alanine	7.35 ± 0.57	7.35 ± 0.65	
Aspartic acid	1.57 ± 0.15	1.12 ± 0.03	*
Glutamic acid	1.68 ± 0.14	1.40 ± 0.08	
Glycine	11.8 ± 0.9	8.71 ± 0.32	*
Isoleucine	2.07 ± 0.11	2.51 ± 0.15	
Leucine	4.24 ± 0.35	7.49 ± 0.54	**
Ornithine	1.61 ± 0.25	1.89 ± 0.14	
Proline	0.873 ± 0.047	0.694 ± 0.242	
Serine	3.60 ± 0.72	2.89 ± 0.29	
Tyrosine	2.34 ± 0.35	2.94 ± 0.30	
Valine	3.92 ± 0.23	4.40 ± 0.35	
Organic acid			
4-Aminobutyric acid	3.00 ± 0.11	2.02 ± 0.06	**
Ethylmalonic acid	97.4 ± 2.0	97.1 ± 2.1	
Malic acid	1.73 ± 0.05	1.47 ± 0.05	*
Phosphoric acid	1235 ± 15	1206 ± 12	
Succinic acid	2.71 ± 0.20	2.00 ± 0.06	*
Others			
2-Hydroxypyridine	9.18 ± 3.16	6.98 ± 0.97	
Glycylglycine	0.866 ± 0.151	1.12 ± 0.13	
Tyramine	0.988 ± 0.096	1.31 ± 0.07	*
Urea	1.50 ± 0.05	1.28 ± 0.04	*

Values are means ± standard errors of the means (SEMs). n = 4/group. The data were analyzed using Student’s *t*-test. *, **: asterisks show significant differences at *P* < 0.05 and 0.01, respectively. The data for glucose, ribose, fructose-6-phosphate, malic acid, and succinic acid are the same as the values in Fig 6.

## 4. Discussion

In this study, we investigated carbohydrate metabolism in ExHC rats by using three types of experimental diets. Through feeding of the high-starch or high-sucrose diets, the ExHC rats developed a type-2-diabetes-like status, high plasma insulin levels, and high HOMA-IR. In addition, the ExHC rats showed low *Pfkl* mRNA levels, responsible for one of two rate-limiting enzymes for glycolysis, in the liver. These results suggest that the ExHC rats have impaired carbohydrate metabolism, especially glucose metabolism. We fed the high-fructose diet to ExHC rats and ExHC.BN-*Dihc2*^*BN*^ congenic rats to evaluate the effect of skipping rate-limiting reactions in glycolysis with high dietary fructose. As we have reported, the ExHC rats show significantly lower hepatic TAG levels than the SD rats, the original strain of ExHC rats, and the Congenic rats then develop DIHC [14,15]. In experiment 2 after consumption of the high-fructose diet, the ExHC rats showed serum cholesterol levels and hepatic TAG contents similar to the Congenic rats. The changes of these DIHC-leading phenotypes mean that ExHC rats do not develop DIHC under high-fructose diet. This study reveals that *Smek2* dysfunction in the ExHC rats causes impairment of glycolysis and this hepatic glucose unavailability leads to the impairment of *de novo* fatty acid synthesis and DIHC.

In our previous studies, we identified *Smek2* as one of the genes responsible for DIHC in ExHC rats [15], and we revealed that *Smek2* dysfunction causes impairment of *de novo* fatty acid synthesis in the liver of ExHC rats to develop DIHC via β-migration of secreted VLDL [15,16]. High sucrose diet is known to activate hepatic fatty acid synthesis [25]. According to these information, we hypothesized that ExHC rats have some disorders in the regulation of FAS activity with sucrose or fructose. In this study, we evaluated glucose metabolism in ExHC rats by feeding them diets rich in sucrose or starch (Experiment 1). After 2 weeks of feeding, the ExHC rats developed higher HOMA-IR, and higher fasting blood insulin levels, despite the diets. These data speculate ExHC rats may develop insulin resistance. On the other hand, the differences between the strains with respect to the final serum fasting glucose levels were significant but not so large (10–20 mg/dL). The glycogen accumulation was observed in the liver of ExHC rats, not in the femoral muscle. Additionally, ExHC rats showed the high fasting blood glucose and quick clearance of blood glucose in the OGTT, especially under the high-starch diet. In OGTT, decreasing rate of blood glucose after peak time is determined with the glucose uptake by peripheral muscles, which is stimulated by insulin. These data suggest that the livers of the ExHC rats have impaired glycolysis and glucose utilization but no impairment in glucose uptake from the blood by peripheral muscle. This insulin resistance in the ExHC rats is speculated to be attributable to hepatic glucose accumulation. The increase in hepatic glycogen in the ExHC rats means that hepatic glucose-6-phosphate is abundant as a substrate for glycogen synthesis and as a metabolic intermediate of glycolysis. Therefore, we focused on the impairment in glycolysis, and evaluated the hepatic expressions of a rate-limiting gene of glycolysis, *Pfkl*. Hepatic *Pfkl* mRNA levels in the ExHC rats were significantly decreased when compared with the SD and Congenic rats. This suggests a slowdown of the entire glycolysis. Therefore, we thought that the hypothesis in Experiment 1 “ExHC rats have a disorder in the sucrose utilization” is not appropriate, and we set up another hypothesis “the primary disorder caused by *Smek2* dysfunction in ExHC rats is a disorder in the glucose utilization in the liver” and conducted Experiment 2.

In order to evaluate the dietary fructose catabolism in ExHC rats, we fed the high-fructose diet to ExHC rats and ExHC.BN-*Dihc2*^*BN*^ congenic strain in experiment 2. This congenic strain has the Dihc2 region, containing *Smek2*, derived from BN rats and background genome derived from ExHC rats. Therefore, pathological phenotypes related to the development of DIHC are ameliorated in the Congenic rats compared with ExHC rats [15]. After the high-fructose diet was fed to the rats, the ExHC and Congenic rats showed similar serum and hepatic lipid levels. In our previous study [16] and Experiment 1 in this study, we confirmed that the hepatic FAS activity of the ExHC rats is low under the high-sucrose and high-starch diet. Whereas the high-fructose diet did not suppress FAS in the Congenic rats, but activated FAS in the livers of the ExHC rats in Experiment 2. Thus, the direct cause of low activities of *de novo* fatty acid synthesis in the livers of the ExHC rats is considered the disorder in hepatic glucose metabolism and shortage of substrate for FAS, not direct dysregulation of FAS gene expression by *Smek2* dysfunction. This is supported by the decrease in malate and succinate, those are the intermediate metabolites in TCA cycle, in the primary hepatocytes of the ExHC rats. In contrast, the increase in cellular glucose and fructose-6-phosphate obviously speculates the slow *Pfkl* activity in the ExHC rats. Hepatic glucose metabolism impairment is responsible for other sequential metabolic impairments in ExHC rats.

The serum cholesterol levels observed in Experiment 2 (ExHC: 474 mg/dL, congenic: 477 mg/dL) were higher than those in the SD rats in Experiment 1 (high-sucrose diet: 111 mg/dL, high-starch diet: 189 mg/dL). The high-fructose diet leads to an increase in serum cholesterol levels via the promotion of hepatic cholesterol synthesis [26]. Indeed, in both strains of experiment 2, liver cholesterol levels, which was greatly higher compared with that of experiment 1, clearly represented that. This phenomenon is confirmed in several rat models [27,28] and is also known to be similar in humans [29]. According to this evidence, high serum cholesterol levels in Experiment 2 are considered to be the side-effect of the high-fructose diet.

The mechanism underlying *Smek2* regulation of *Pfkl* mRNA expression in the liver is still unclear. On the basis of the results of the water-soluble metabolite analysis, the increase in ribose in the primary hepatocytes of the ExHC rats can be a clue for elucidating the mechanism. This increase in ribose can be interpreted as the result of increased glucose influx to the pentose-phosphate pathway due to low *Pfkl* expression in the liver. A similar phenomenon was reported in cancer cells. Cancer cells under carbon monoxide (CO) stress suppress PFK activity and perform glycolysis through the pentose-phosphate pathway for proliferation [30]. The regulation of PFK activity in CO-stressed cancer cells is through methylation of a PFK activator protein [30]. Methylation of the *Pfk* promoter region has been reported to be involved in the regulation of *Pfk* mRNA expression [31,32]. The low *Pfkl* expression caused by *Smek2* dysfunction in the ExHC rats may be mediated by an abnormality in the methylation enzyme or an abnormality in the supply pathway of the methyl group. There is also an important link between the methylation status of fructokinase-related genes and carcinogenesis. According these report, further study is necessary to reveal the mechanism how *Smek2* disorder leads low hepatic *Pfkl* expression.

In this study, we confirmed the significant increase in brain weights by the consumption of the high-starch diet compared with the high-sucrose diet, and the significant decrease in the brain weight of the ExHC rats when compared with those of the SD rats in this study. The relationship between dietary carbohydrates and brain function has been the subject of many years of research [33,34]. Further studies on the brain functions of ExHC rats may provide useful information.

## 5. Conclusion

*Smek2* dysfunction causes an impairment of glucose utilization. This impairment weakens the ability to synthesize fatty acids in the livers of ExHC rats. These sequential disorders result in DIHC in ExHC rats.

## Supporting information

S1 Table Diet compositions. (DOCX)

S2 Table Composition of EGTA solution for primary hepatocyte separation. (DOCX)

S3 Table Composition of collagenase solution for primary hepatocyte separation. (DOCX)

S4 Table Composition of Hanks solution for primary hepatocyte separation. (DOCX)

## List of abbreviations

ExHC rat: Exogenously hypercholesterolimic rat
SD rat: Sprague-Dawley rat
OGTT: oral glucose tolerance test
HOMA-IR: homeostasis model assessment as an index of insulin resistance
TAG: triacylglycerol
NEFA: non-esterified fatty acid
ANOVA: analysis of variance
