# Supplementary material for: Low utilization of glucose in the liver causes diet-induced hypercholesterolemia in exogenously hypercholesterolemic rats
Source: PLoS One. 2020 Mar 12;15(3):e0229669. doi: 10.1371/journal.pone.0229669 (PMC7067558; doi:10.1371/journal.pone.0229669)
Supplement: S1 Table — (DOCX) [file pone.0229669.s001.docx]

**S1 Table. Diet compositions.**
