# Supplementary material for: Low utilization of glucose in the liver causes diet-induced hypercholesterolemia in exogenously hypercholesterolemic rats
Source: PLoS One. 2020 Mar 12;15(3):e0229669. doi: 10.1371/journal.pone.0229669 (PMC7067558; doi:10.1371/journal.pone.0229669)
Supplement: S4 Table — (DOCX) [file pone.0229669.s004.docx]

**S4 Table. Composition of Hanks solution for primary hepatocyte separation.**

The pH of the solution was adjusted to 7.6 with NaOH, and the solution was sterilized with a PVDF filter (0.22 μm). The solution was stored at 4 °C until use.
